# Suppression of Plant Defenses by Herbivorous Mites Is Not Associated with Adaptation to Host Plants

**DOI:** 10.3390/ijms19061783

**Published:** 2018-06-15

**Authors:** Jéssica T. Paulo, Diogo P. Godinho, Anabela Silva, Cristina Branquinho, Sara Magalhães

**Affiliations:** 1 cE3c Centre for Ecology, Evolution and Environmental Changes, Faculdade de Ciências, Universidade de Lisboa, 1649-004 Lisboa, Portugal; jessicapaulo94@gmail.com (J.T.P.); diogoprinogodinho@gmail.com (D.P.G.); cmbranquinho@fc.ul.pt (C.B.); 2 BioISI, Biosystems and Integrative Sciences Institute, Faculdade de Ciências, Universidade de Lisboa, 1649-004 Lisboa, Portugal; arsilva@fc.ul.pt

**Keywords:** plant-herbivore interactions, solanales, tetranychidae, host-plant adaptation

## Abstract

Some herbivores suppress plant defenses, which may be viewed as a result of the coevolutionary arms race between plants and herbivores. However, this ability is usually studied in a one-herbivore-one-plant system, which hampers comparative studies that could corroborate this hypothesis. Here, we extend this paradigm and ask whether the herbivorous spider-mite *Tetranychus evansi*, which suppresses the jasmonic-acid pathway in tomato plants, is also able to suppress defenses in other host plants at different phylogenetic distances from tomatoes. We test this using different plants from the Solanales order, namely tomato, jimsonweed, tobacco, and morning glory (three Solanaceae and one Convolvulaceae), and bean plants (Fabales). First, we compare the performance of *T. evansi* to that of the other two most-commonly found species of the same genus, *T. urticae* and *T. ludeni*, on several plants. We found that the performance of *T. evansi* is higher than that of the other species only on tomato plants. We then showed, by measuring trypsin inhibitor activity and life history traits of conspecific mites on either clean or pre-infested plants, that *T. evansi* can suppress plant defenses on all plants except tobacco. This study suggests that the suppression of plant defenses may occur on host plants other than those to which herbivores are adapted.

## 1. Introduction

Plants have a broad set of defenses that allow them to decrease their susceptibility to herbivore attack [1]. This may result in the decrease of food quality to herbivores, reducing their survival and fecundity [2]. Herbivores, in turn, have evolved diverse strategies to circumvent plant defenses, allowing them to counter the negative effects caused by such defenses and maximize the conversion of plant material into offspring [3]. Such a history of evolution of defense and counter-defense may lead to a plant–herbivore coevolutionary arms race [4].

There is ample variation in how herbivores cope with defensive plant traits. Whereas some species have evolved means to avoid tissues with high levels of toxic compounds [5,6,7] or to digest them [8,9,10], others can manipulate the induction of plant defenses by fully or partially suppressing them [11,12,13,14,15,16]. Suppression was found in several plant pathogens and insects, being often associated with an increase of herbivore performance [11,15,16,17,18,19].

This ability to suppress plant defenses is generally believed to result from plant-herbivore coevolution. If so, then one would expect suppression to be expressed on plants to which herbivores are adapted to (or in phylogenetically close plants), and not on other plants. Most herbivore-plant defensive interactions have been traditionally addressed using one herbivore and one plant species [20]. Some studies, however, have moved beyond this paradigm by using a few plant species or several herbivores and one plant species [16,21,22,23]. However, these studies concern herbivores that induce, not suppress, plant defenses, with the exception of Godinho et al. (2016), which compares the suppression of two spider mite species on tomato. Clearly, studies addressing how the ability to suppress plant defenses extends to different host plants are highly needed.

Spider mite species of the genus *Tetranychus* are important crop pests that feed by piercing leaf mesophyll cells and sucking the intracellular content [24,25]. Within this group, *T. urticae* induces tomato plant (*Solanum lycopersicum*) defenses [12,15,26,27,28]. On these plants, as with many others, after herbivore feeding, genes that downstream the jasmonic acid (JA) biosynthesis are induced [26,29,30]. This induction leads to an increase in the expression of wound-induced cysteine- and serine-like proteinase inhibitors [26], which affect herbivore performance by interfering with their digestion and their offspring development, respectively [31]. However, some populations of *T. urticae* were found to suppress such tomato plant defenses [12]. Suppression was also found in *T. evansi* and *T. ludeni* [15,16,32]. Those species may even suppress plant defenses to levels below those of non-attacked plants, although this ability was not found in all spider mite populations [32].

Although the interaction between *T. evansi* and tomato defenses is well-studied, little is known about how this ability extends to other plants, namely those belonging to the Solanales order, to which the tomato belongs. In the present study, we aim to fill this gap by testing whether *T. evansi* also suppresses the defenses of other plant species from the Solanaceae and Convolvulaceae families, namely tomato, jimsonweed, tobacco, and morning glory, and bean plants (Fabales). We chose tomato because it is the host plant on which suppression has been identified [15], and bean because it is a plant commonly used by spider mites and unrelated to tomato. The other host plants were chosen based on (a) their phylogenetic distance to tomato, (b) the fact that plant-herbivore interactions and their chemical blend of secondary metabolites are well studied [33], (c) spider mites have been found on those plants [34], and (d) they are relatively easy to rear in the laboratory. First, we address the degree of local adaptation of *T. evansi*. This was performed by comparing its performance, on each host plant, to that of two other species of the same genus, *T. urticae* and *T. ludeni*, which are often found in the same locations as *T. evansi* in Southwestern Europe [35]. Second, the effect of *T. evansi* on plant defenses was evaluated by pre-infesting plants with this mite species and subsequently analyzing conspecific performance and plant physiological measurements. Specifically, we measured leaf reflectance between 300.4 nm and 313.9 nm which correspond to the area of UV-light, as it has been shown that herbivores affect plants similarly to UV-B light exposure [36,37]. Therefore, differences in leaf reflectance at the spectra of 300.4 nm to 313.9 nm, between clean and pre-infested plants, indicate herbivore effects on plants. Additionally, wound inducible trypsin-like inhibitor (TI) activity has been validated as a good indicator of the level of expression of plant defenses in several host plants and against several herbivores [23,38,39,40,41,42], including those of tomato plants against spider mites [15,16,26,31]. TIs inhibit the digestive serine proteases of several organisms and are inducible by JA and wounding (i.e., mite feeding) in several plant species [43,44,45]. Thus, to assess the effect of spider mites on the plant defenses, TI activity was measured. We showed that *T. evansi* can suppress plant defenses on the plant to which it is locally-adapted (tomato). However, it also suppresses the defenses of other plants, including those of distantly-related plants such as the bean. This study revealed that suppression may not be systematically associated to herbivores being adapted to their host plants.

## 2. Results

### 2.1. Performance of Tetranychus Species on Several Host Plants

The survival of females from the three spider mite species, on leaf discs, was differentially affected by the different host plants (Figure S1; Interaction host plant × mite species, $X_{8}^{2}$ = 32.83, *p* < 0.001, Table S1). Analyses within each plant species showed that all mite species had similar survival rates on tomato ($X_{2}^{2}$ = 3.328, *p* = 0.189), jimsonweed ($X_{2}^{2}$ = 4.547, *p* = 0.103) and bean ($X_{2}^{2}$ = 3.476, *p* = 0.176) plants. However, *T. evansi* females had higher survival rates than *T. ludeni* on tobacco plants (*T. evansi* vs. *T. ludeni*: *Z* = −2.639, *p* = 0.022), but lower survival rates than *T. ludeni* on morning glory plants (*T. evansi* vs. *T. ludeni*: *Z* = 2.752, *p* = 0.015), whereas other comparisons revealed non-significant differences (Figure S1; Table S1).

**Figure 1:**
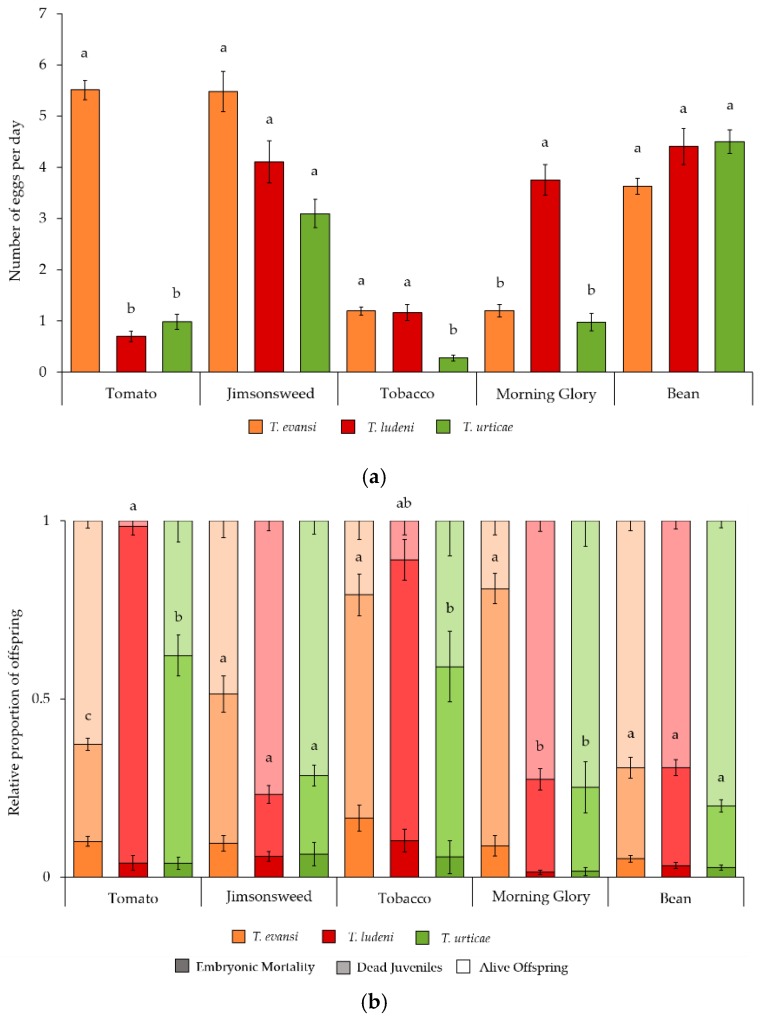
Performance *of T. evansi*, *T. ludeni* and *T. urticae* on tomato, jimsonweed, tobacco, morning glory or bean leaf discs: (**a**) Average (± standard error) oviposition rate; (**b**) Average (± standard error) proportion of embryonic mortality, dead juveniles, and alive offspring (bottom to top). Different letters indicate significant differences in oviposition rate (**a**) and dead juveniles (**b**) among mite species within plant species.

The oviposition rate (Figure 1a) was also significantly affected by the interaction between a mite and host plant species (*F*_8654.4_ = 62.19, *p* < 0.001). Tukey post-hoc comparisons within plant species revealed that the three-mite species had a similar oviposition rate on jimsonweed (*F*_23.807_ = 1.585, *p* = 0.316) and bean plants (*F*_216.70_ = 1.233, *p* = 0.317). *T. evansi* had the highest fecundity on tomato plants (*T. evansi* vs. *T. ludeni*: *Z* = 18.33, *p* < 0.001; *T. evansi* vs. *T. urticae*: *Z* = 17.15, *p* < 0.001; *T. ludeni* vs. *T. urticae*: *Z* = −1.209, *p* = 0.447). Conversely, *T. ludeni* had the highest fecundity on morning glory plants (*T. evansi* vs. *T. ludeni*: *Z* = 8.611, *p* < 0.001; *T. evansi* vs. *T. urticae*: *Z* = 0.651, *p* = 0.792; *T. ludeni* vs. *T. urticae*: *Z* = 9.596, *p* < 0.001). Additionally, on tobacco plants, both *T. evansi* and *T. ludeni* had higher fecundity than *T. urticae*, with no significant differences between them (*T. evansi* vs. *T. ludeni*: *Z* = 0.939, *p* = 0.615; *T. evansi* vs. *T. urticae*: *Z* = 7.127, *p* < 0.001; *T. ludeni* vs. *T. urticae*: *Z* = 7.033, *p* < 0.001).

Embryonic mortality (Figure 1b) was significantly affected by the plant (*F*_4679_ = 8.673, *p* < 0.001), the mite species *F*_2679_ = 6.383, *p* = 0.002), but not by their interaction ($X_{8}^{2}$ = 7.495, *p* = 0.484). The plant species, mite species, and their interaction significantly affected juvenile mortality (Figure 1b; *F*_4670_ = 43.80, *p* < 0.001; *F*_2670_ = 3.346, *p* = 0.036; and *F*_8670_ = 30.85, *p* < 0.001, respectively). Analysis within plant species showed similar juvenile mortality on jimsonweed (*F*_297_ = 0.372, *p* = 0.690) and bean (*F*_2148_ = 1.073, *p* = 0.345) plants. Additionally, *T. evansi* had the lowest juvenile mortality on tomato (*T. evansi* vs. *T. ludeni*: *Z* = −7.729, *p* < 0.001; *T. evansi* vs. *T. urticae*: *Z* = −6.278, *p* < 0.001), whereas it was the highest on morning glory plants (*T. evansi* vs. *T. ludeni*: *Z* = 6.521, *p* < 0.001; *T. evansi* vs. *T. urticae*: *Z* = 6.426, *p* < 0.001). On tobacco plants, *T. evansi* had higher juvenile survival than *T. urticae* (*T. evansi* vs. *T. urticae*: *Z* = 2.490, *p* = 0.033), with other comparisons yielding non-significant results (Table S1).

### 2.2. The Effect of T. evansi Infestations on Plant Defences

#### 2.2.1. Reflectance Spectroscopy Analysis

**Figure 2:**
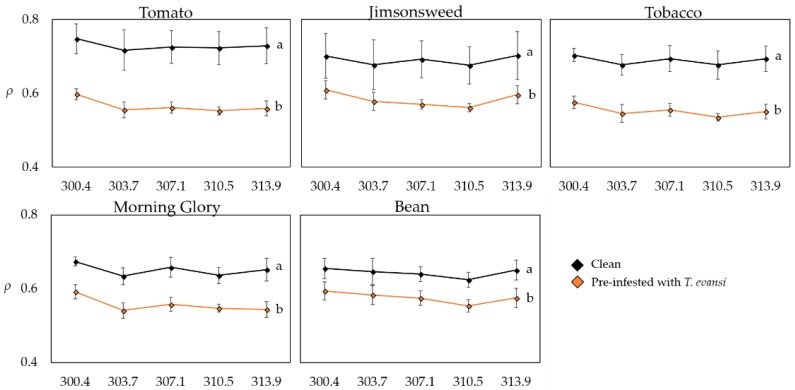
Effect of plant species and infestation status on the leaf reflectance factor (*ρ*) on wavelengths between 300.4 nm and 313.9 nm. The markers represent the average (± standard error) of *ρ* for each plant species, either clean or pre-infested by *T. evansi*. Different lowercase letters indicate significant differences in *ρ* among clean plants and plants pre-infested with *T. evansi*, within plant species.

Leaf reflectance (Figure 2) varied significantly with the plant species in the wavelengths of 310.5 nm (*F*_447.47_ = 3.178, *p* = 0.022) and 313.9 nm (*F*_447.53_ = 2.625, *p* = 0.046; Table S2), and infestation status had a significant effect in all wavelengths tested (300.4 nm: *F*_150.10_ = 71.65, *p* < 0.001; 303.7 nm: *F*_150.08_ = 50.57, *p* < 0.001; 307.1 nm: *F*_150.28_ = 52.43, *p* < 0.001; 310.5 nm: *F*_146.09_ = 87.30, *p* < 0.001; 313.9 nm: *F*_146.10_ = 60.08, *p* < 0.001). There was no significant interaction between plant species and infestation status in all wavelengths tested (*p* > 0.05, Table S2). In fact, all plants pre-infested with *T. evansi* had significantly lower spectral reflectance factors (*ρ*) in all the studied wavelengths.

#### 2.2.2. Trypsin Inhibitors Quantification

**Figure 3:**
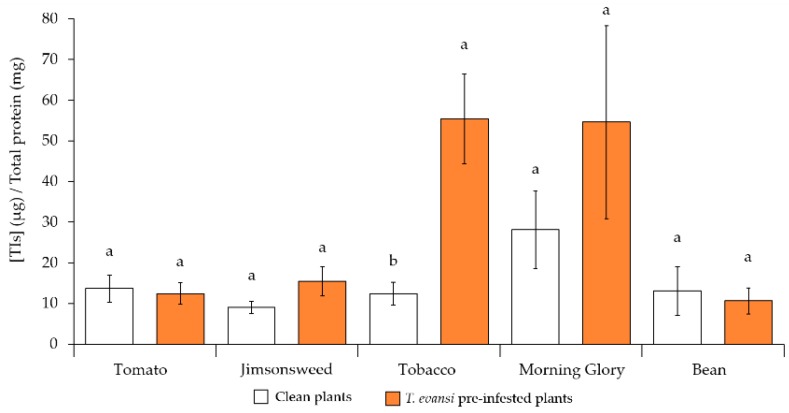
Effect of the infestation status (clean plants or plants pre-infested with *T. evansi*) on therelative content of trypsin inhibitors (TIs, µg mg^−1^ total soluble protein) on tomato, jimsonweed, tobacco and bean plants. Different letters indicate significant differences in the average (±standard error) relative content of TIs among infestation status within plant species.

The relative content of TIs (µg) per total soluble protein (mg) present on each sample was significantly affected by the interaction between plant species and infestation status (Figure 3; *F*_447.83_ = 3.373, *p* = 0.041). Analysis within plant species showed that the relative content of TIs was similar between clean and *T. evansi* pre-infested plants on tomato (*F*_18.835_ = 0.003, *p* = 0.960), jimsonweed (*F*_16.988_ = 2.916, *p* = 0.132), morning glory (*F*_14.888_ = 0.097, *p* = 0.768), and bean (*F*_15.370_ = 0.305, *p* = 0.603) plants. These results suggest that *T. evansi* was able to suppress TIs to levels of clean plants on these plant species. Additionally, there was an increase in the relative content of TIs on tobacco plants pre-infested with *T. evansi* (*F*_18.102_ = 12.92, *p* = 0.007), suggesting the induction of TIs in this plant species.

#### 2.2.3. Conspecific Performance

During infestation, the mortality of *T. evansi* females was significantly affected by the host plant (*F*_4,29_ = 15.08, *p* < 0.001, Figure S2). Indeed, infesting females survive less on morning glory than on all other plants, on which their rates of survival are similar (jimsonweed-bean: *Z* = 2.620, *p* = 0.058; tobacco-bean: *Z* = 1.588, *p* = 0.463; tomato-bean: *Z* = 0.540, *p* = 1.000; tobacco-jimsonweed: *Z* = −0.571, *p* = 0.974; tomato-jimsonweed: *Z* = −0.744, *p* = 0.934; tomato-tobacco: *Z* = −0.164, *p* = 1.000, morning glory-bean: *Z* = −6.828, *p* < 0.001; morning glory-jimsonweed: *Z* = −6.525, *p* < 0.001; morning glory-tobacco: *Z* = −5.676, *p* < 0.001; morning glory-tomato: *Z* = −5.818, *p* < 0.001).

On the leaf discs, the survival of *T. evansi* conspecific females was not significantly different on clean or *T. evansi* pre-infested plants (${\;X}_{1}^{2}$ = 1.221, *p* = 0.269, Figure S3), and this result was not affected by the plant species (interaction between the plant species and infestation status:${\;X}_{4}^{2}$ = 6.011, *p* = 0.198).

**Figure 4:**
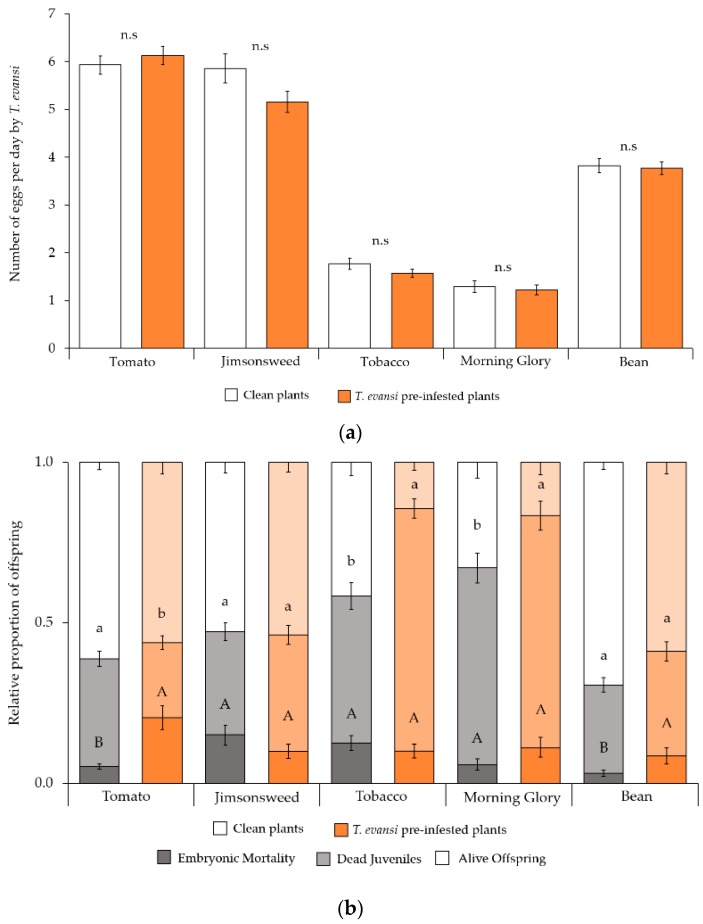
Performance of *T. evansi* on tomato, jimsonweed, morning glory, or bean leaf discs, either clean or pre-infested by conspecifics: (**a**) Average (± standard error) oviposition rate; and (**b**) Average (±standard error) proportion of unhatched eggs, dead juveniles, and alive offspring (bottom to top). n.s indicates non-significant differences on oviposition rate (**a**) among spider mites within plant species. Different uppercase letters indicate significant differences in the proportion of unhatched eggs (**b**) among mite species within plant species. Lowercase letters indicate significant differences in dead juveniles (**b**) among mite species within plant species.

Plant species significantly affected the oviposition rate of *T. evansi* females (*F*_4609.2_ = 367.9, *p* < 0.001; Figure 4a). However, this trait was not significantly affected by either infestation status or its interaction with the plant species (*F*_1651.3_ = 0.879, *p* = 0.349; *F*_4647.8_ = 0.807, *p* = 0.521, respectively). These results suggest that no cost or benefit was conferred to conspecifics by pre-infestation with *T. evansi*.

The proportion of unhatched eggs (Figure 4b) was significantly affected by the plant, infestation status, and their interaction (*F*_4636_ = 15.044, *p* < 0.001; *F*_1636_ = 4.373, *p* = 0.037; *F*_4636_ = 3.580, *p* = 0.007, respectively). Further analysis within plant species revealed that, on tomato and bean plants pre-infested by *T. evansi*, the proportion of unhatched eggs was higher than on clean plants (tomato: *F*_1153_ = 13.09, *p* < 0.001; bean: *F*_1117_ = 4.485, *p* = 0.034). On jimsonweed, tobacco, and morning glory plants, however, embryonic mortality was not affected by the infestation status (jimsonweed: *F*_1114_ = 1.499, *p* = 0.221; tobacco: *F*_1139_ = 3.593, *p* = 0.058; morning glory: *F*_1105_ = 0.268, *p* = 0.606).

Overall, the proportion of dead juveniles (Figure 4b) was similar between clean and *T. evansi* pre-infested plants (*F*_1636_ = 0.677, *p* = 0.411). However, plant species and the interaction between plant and mite species significantly affected this proportion (*F*_4636_ = 11.05, *p* = 0.044; *F*_4636_ = 9.617, *p* < 0.001, respectively). Analysis within plant species showed that the proportion of dead juveniles was lower for tomato plants pre-infested by *T. evansi* (*F*_1153_ = 8.626, *p* = 0.003) and higher for tobacco and morning glory plants pre-infested with *T. evansi* (tobacco: *F*_1139_ = 36.30, *p* < 0.001; morning glory: *F*_1105_ = 4.715, *p* = 0.032). Moreover, the proportion of dead juveniles on jimsonweed and bean plants was similar on clean plants or plants pre-infested with *T. evansi* (jimsonweed: *F*_1114_ = 1.446, *p* = 0.229; bean: *F*_1117_ = 0.702, *p* = 0.402).

The total offspring mortality (Figure 4b) was significantly affected by plant, mite species, and their interaction (*F*_4636_ = 29.19, *p* < 0.001; *F*_1636_ = 4.677, *p* = 0.031; *F*_4636_ = 4.335, *p* = 0.002, respectively). Analysis within plant species revealed that the total offspring mortality was similar on pre-infested tomato and jimsonweed plants (tomato: *F*_1153_ = 3.283, *p* = 0.072; jimsonweed: *F*_1114_ = 1.874, *p* = 0.179) compared to clean plants. For tobacco, morning glory, and bean plants pre-infested with *T. evansi*, the total mortality was higher than that on the respective clean plants (tobacco: *F*_1139_ = 31.53, *p* < 0.001; morning glory: *F*_1105_ = 6.291, *p* = 0.014; bean: *F*_1117_ = 7.011, *p* = 0.009).

## 3. Discussion

In this study, we show that the performance of *T. evansi* is higher than that of the other mite species on tomato plants, in terms of both fecundity and offspring mortality. However, this is not the case for the other host plants. Therefore, this mite species exhibits a pattern of local adaptation [46] on tomato plants. Moreover, for all plants except tobacco, we found no differences in TIs levels on clean plants vs. plants pre-infested with *T. evansi*. Because the analysis of leaf reflectance showed that mites modified the state of the plant, we may conclude that these similar levels of TIs were due to *T. evansi* suppressing plant defenses on all plants except tobacco. However, TIs levels were not always associated with conspecific performance.

Our life-history traits data, on clean plants that include other spider mite species, allowed us to conclude that *T. evansi* is locally adapted [46] to tomato plants. Moreover, on morning glory plants, the performance of *T. ludeni* was significantly higher compared to that of *T. evansi* and *T. urticae*, suggesting local adaptation [46] of this mite species on that host plant. Additionally, while the performance of all spider mite species was relatively high on jimsonweed and bean plants, it was quite low on tobacco plants. This may be due to the high levels of nicotine present in tobacco leaves, which can serve as a constitutive defense for spider-mites as well as for other herbivores [47]. However, differences in mite performance across host plants may also be partly due to effects of the host plant on which spider mites have been reared and/or due to maternal effects [48].

Plants pre-infested with *T. evansi* had a lower leaf spectral reflectance factor, on the 300.4 nm to 313.9 nm spectra, than clean plants. This is an important result, because on some host plants, such as morning glory and tobacco, *T. evansi* life-history traits had low values. We could thus question whether herbivores were interacting at all with the plant, in which case we would find no differences of TI activity between clean and pre-infested plants. From the leaf reflectance analysis, we can conclude that mites are actively interacting with all host plants. Addressing which type of interaction gives way to these reflectance patterns requires further analysis.

On tomato, jimsonweed, and bean plants, we found suppression of TI activity, as this activity did not differ between clean or pre-infested plants. However, TI activity in pre-infested plants was never below the basal levels of clean plants. As found in previous studies [15,16], a similar result regarding tomato plants was observed for other populations of *T. evansi* [32]. Therefore, there seems to be population variability for suppression levels. This may be due to differences in the evolutionary history of the populations (geographic location, host plant, among others.), or to different selection pressures in different laboratories.

Overall, we found a reasonable association between the effect of mite infestation on TI levels and on mite life-history traits, with two exceptions: On tobacco we found induction of TIs but no effect on the oviposition rate on this plant, whereas on morning glory we found suppression of TIs but an increase in juvenile mortality. The first result may be explained by the fact that oviposition on clean tobacco plants was very low, potentially hampering the detection of differences between treatments. Concerning the second result, female mortality during the infestation protocol was significantly higher on morning glory than on the other plants, possibly leading to a lower effect of herbivory. This may explain the high variation across replicates for TI activity of pre-infested morning glory plants. Despite the significant effect of *T. evansi* on morning glory plants observed through the leaf reflectance assay, the high mortality of infesting females may have prevented the detection of a significant induction of TIs on this host plant. To corroborate this hypothesis, plants could be infested with higher densities of spider mites to compensate for high mortality.

On tobacco and morning glory plants, but not on the other plants, *T. evansi* pre-infestations led to an increase in juvenile mortality, compared to clean plants. This may be associated with the induction of TI activity on tobacco plants and the probable induction on morning glory plants. It was previously shown that serine proteases, including trypsin and chymotrypsin-like proteases, are essential to the development of spider mites [31]. Indeed, after feeding on plants on which inhibitors for these proteases (TIs) are induced, spider mite juvenile development can be delayed or even arrested, leading to an increase in juvenile mortality [31]. In the current study, this trait seems to be well related with the effect of spider mites on TI activity. The other life-history traits, oviposition and female survival, are expected to be more affected by cysteine-like proteinase inhibitors, which affect a spider mite’s digestion by inhibiting the cysteine proteases produced in the mite’s gut [31,49]. Thus, although TIs have been amply validated as good surrogates for the induction of plant defenses by spider mites [15,16,26], measuring the activity of cysteine-like proteinase inhibitors could provide a broader view on the differences in induced defenses across plants.

We found suppression on several plants, but *T. evansi* was only locally adapted to tomato. That is, we found no association between the ability to suppress plant defenses and a pattern of local adaptation. Given that local adaptation generally indicates a long coevolutionary history between plants and herbivores, we here show that no association between suppression ability and a long coevolutionary history is expected. In fact, suppression on other plant species may be a by-product of the adaptation of spider mites to tomato plants defenses. Indeed, several plant species use the same pathway (JA) as a defense against herbivory; hence, a similar response may be expected on other host plants.

From an ecological perspective, our results suggest that *T. evansi* will not benefit from being on plants with conspecifics as compared to being on clean plants. If this holds true under more natural settings, it means that spider mite distribution on plants with or without *T. evansi* will be due to effects associated with resource competition rather than to effects associated with interaction with plant defenses. As this is probably not the case in a landscape in which *T. urticae* appears first (thus inducing plant defenses), it may be interesting to compare the distribution of mites on landscapes with different orders of infestation [50]. Moreover, suppression was also not associated with local adaptation of *T. evansi*. This means that, on plants in which the performance of heterospecifics (*T. ludeni* and *T. urticae*) is similar or even higher than that of *T. evansi*, suppression will probably benefit heterospecifics at least to the same extent as conspecifics. Thus, it is not clear that *T. evansi* collects a net benefit from suppressing defenses on those host plants. Clearly, more studies testing herbivores that suppress plant defenses on several plants are needed, preferably with an accurate control of their recent evolutionary history.

## 4. Materials and Methods

### 4.1. Plants

Four plant species from the Solanales order (Solanaceae and Convolvulaceae families) were used: Tomato (*Solanum lycopersicum*, var. Moneymaker, Johnsons, Suffolk, UK), jimsonweed (*Datura stramonium*, Botanical Garden of University of Trás-os-Montes e Alto Douro, Vila Real, Portugal), tobacco (*Nicotiana tabacum*, var. Virginia, Faculty of Sciences of University of Lisbon, Lisbon, Portugal), and purple morning glory (*Ipomoea purpurea*, Vilmorin, Paris, France). Bean plants (*Phaseolus vulgaris*, var. Contender, Germisem, Oliveira do Hospital, Portugal) from the Fabales order were used as an outgroup of the Solanales order. All plants used were sown and grown in an Aralab climatic chamber under controlled conditions (25–20 °C; 70% RH; photoperiod of 16L:8D). The taxonomic description, the age at which each plant was used, and which leaf was selected for the experiments are summarized in Table S3.

### 4.2. Spider Mite Cultures

*Tetranychus evansi* and *T. ludeni* were collected from jimsonweed plants (*D. stramonium*) in 2013 in Alenquer and Assafora, Portugal, respectively. *T. urticae* was collected in Carregado, Portugal, from tomato plants (*S. lycopersicum*) in 2010. Laboratory populations were formed from 500, 600, and 300 *T. evansi*, *T. ludeni*, and *T. urticae* individuals, respectively. *T. evansi* was maintained on four-weeks-old tomato plants (*S. lycopersicum*, var. Moneymaker), and *T. ludeni* and *T. urticae* were maintained on two-weeks-old bean plants (*P. vulgaris*), over approximately 160 generations. Following this, they were maintained on four-weeks-old tomato plants (*S. lycopersicum*, var. Moneymaker) over approximately 20 generations. All populations were reared in large numbers (>2000) under controlled conditions (25 °C; photoperiod of 16L:8D). Experiments were also performed under those same conditions.

For the present work, *T. ludeni* and *T. urticae* populations were reared on morning glory plants over approximately 10 generations, and the *T. evansi* population was reared on tomato plants over approximately 30 generations.

### 4.3. Performance of Tetranychus Species on Several Host Plants

Leaf discs (11 mm^2^ Ø) were made from non-infested plants (Table S3) and placed in water-saturated cotton. 15 ± 1 days-old mated females from each mite species were added to each leaf disc and allowed to oviposit for 4 days. Every day, the female status (alive, dead, or drowned) was recorded and, on the fourth day, the number of eggs laid was counted. 5 days later, the number of juveniles that hatched from the eggs (i.e., the hatching rate) was measured, and, 7 days later, the number of adults and dead juveniles on each leaf disc was measured (offspring survival).

### 4.4. The Effect of T. evansi Infestations on Plant Defences

Mite infestations were performed by placing 100 *T. evansi* mated females over 72 h on one fully-expanded non-detached leaf of each plant species (Table S3). This leaf was isolated from the rest of the plant by applying Vaseline to the petiole. The females, eggs and web were then removed and the number of live and dead females was assessed (infestation mortality). Afterwards, the performance of conspecific *T. evansi* females was measured, applying the same protocol as in Section 4.3. As a control, clean plants, also treated with Vaseline, were tested in the same controlled conditions (25 °C; photoperiod of 16L:8D) over 72 h.

#### 4.4.1. Reflectance Spectroscopy Analysis

Using a UniSpec spectroradiometer (PP-Systems, Haver Hills, MA, USA), the reflectance spectra of the selected leaves were measured in the range of UV-B light (300.4 nm–313.9 nm) wavelengths, with an optimized integration time of 30 ms. Five measurements were performed per plant replicate (clean or after 72 h of infestation). Spectral reflectance factors (*ρ*) were obtained by normalizing the reflected radiation from the leaves by a reflectance white standard.

#### 4.4.2. Trypsin Inhibitors Quantification

To quantify TI activity, the Kassel protocol [51] was used. Using a Qiagen TissueRuptor (Qiagen, Hilden, Germany), ~300 mg of the plant material (either pre-infested or clean plants), previously stored at −80 °C, was ground and homogenized with 600 μL of extraction buffer (0.1 M Tris-HCl, pH 8.2; 20 mM CaCl_2_; 1:3). After centrifuging each sample at 4 °C, 16,000× *g* for 25 min, the supernatant was separated from the pellet and maintained at 4 °C during the entire procedure (to preserve the sample from autohydrolysis). Immediately before use, a trypsin solution containing 13,000–20,000 units/mL of trypsin and a 0.1% (*w*/*v*) *N*-Benzoyl-d,l-arginin-4 nitroamilide hydrochloride (BApNA) solution was prepared. To quantify TI activity through trypsin inhibition, several measures were defined: (1) “Positive control” for the full hydrolytic activity of trypsin upon BApNA in the absence of an inhibitor (15 µL of trypsin + 135 µL of extraction buffer + 75 µL of BApNA); (2) “BApNa negative” control for the autohydrolysis of trypsin (15 µL of trypsin + 210 µL of extraction buffer); (3) “Trypsin negative” control for the autohydrolysis of BApNa (150 µL of extraction buffer + 75 µL of BApNA); (4) “Sample control” for the hydrolytic activity intrinsic to the sample and for the supernatant color (135 µL of extraction buffer + 15 µL of sample + 75 µL of BApNA); (5) “Sample” quantification assay per se, in which the hydrolytic activity of trypsin is partially inhibited by the TIs present in the plant extracts (15 µL of trypsin + 120 µL of extraction buffer + 15 µL of sample + 75 µL of BApNA).

In a 96 well plate, each of the treatments described above was performed in triplicate. The plate was incubated 10 min at room temperature, and then BApNA was added. Immediately afterwards, the absorbance was read in a micro-plate reader (BioTek, Winooski, VT, USA) at 405 nm (*t* = 0 min) and was left to incubate at room temperature for another 5 min. After that, the absorbance was read again (*t* = 5). The proportion of trypsin inhibition was calculated as the difference between the two readings (Δ) (1).

(1) $$\mathrm{Inibibition}\;=\;1\;-\;\left[\frac{\Delta \mathrm{Sample}\;-\;\left(\Delta \mathrm{Sample}\;\mathrm{control}\;+\;\Delta \mathrm{Trypsin}\;\mathrm{negative}\;+\;\Delta \mathrm{BApNA}\;\mathrm{negative}\right)}{\Delta \mathrm{Positive}\;\mathrm{control}\;-\;\left(\;\Delta \mathrm{Trypsin}\;\mathrm{negative}\;+\;\Delta \mathrm{BApNA}\;\mathrm{negative}\right)}\right]$$

To determine the TI concentration that leads to inhibition, the relative quantification of total soluble protein present in each sample was determined by the Bradford method [52], using bovine serum albumin (BSA) as standard. Due to technical constraints, the spectrophotometry readings were performed at 630 nm and not at 595 nm. However, the accuracy and resolution of the method at this wavelength was tested and verified (Figure S4).

As the trypsin solution was known to have 100 μg/mL of protein, the relative content of TIs in each sample was expressed by total soluble protein content in leaf extract (2).

(2) $$\left[\mathrm{TIs}\right]\;=\;\frac{\mathrm{Inhibition}\;\times \;10^{5}}{\left[\mathrm{Total}\;\mathrm{protein}\right]}{\;\mu g\;\mathrm{mg}}^{-1}\;\mathrm{protein}$$

Subsequently, the performance of 15 ± 1 days-old conspecific mated females on clean and pre-infested plants was measured as described in Section 4.3. This provides a reliable measure of the effect of defense induction or suppression on mite performance.

#### 4.4.3. Conspecific Performance

Performance of conspecifics on clean and pre-infested plants was determined as described in Section 4.3. (*Performance of Tetranychus species on several host plants*).

### 4.5. Statistical Analysis

All statistical analyses were performed with the software R (version 3.2.5, R Development Core Team 2016, Chichester, UK). The description of the statistical models is available in Table S4.

To analyze the data regarding Section 2.1 (*Host range of Tetranychus species*), plant and mite species were defined as fixed explanatory variables, and block was defined as a random explanatory variable. For the data of Section 2.2 (*The effect of T. evansi infestations on plant defenses*), plant species and infestation status were defined as fixed explanatory variables and block as a random explanatory variable.

To understand the differences among mite species within plant species (Section 2.1 *Performance of Tetranychus species on several host plants*) fTukey post-hoc comparisons, applied to each plant subset, were done using a General Linear Hypothesis Test (glht, multcomp package) [53]. To compare the effect of infestation status within plant species (Section 2.2 *The effect of T. evansi infestations on plant defenses*), in the cases where a significant interaction between plant species and infestation status was found, the significance of infestation status was tested for each plant species separately.

To analyze female survival over the 4-day oviposition period, a cox proportional hazard mixed-effect model was used (coxme, coxme package) [54], with accidental deaths (i.e., drowned females and live females disposed of at the fourth day) as censored. For the subsequent analyses, the females that died accidentally before the fourth day were excluded.

For the analysis of oviposition rate, Box-cox transformations [55] were performed to improve normality (Section 2.1 *Performance of Tetranychus species on several host plants*: λ = 0.154; Section 2.2 *The effect of infestations by T. evansi on plant defenses*: λ = 0.265) and linear mixed-effect models (lm, lme4 package) were used [56].

To test for differences in the proportion of unhatched eggs, dead juveniles and live offspring, the three explanatory variables were computed using the cbind function. For the data of Section 2.2 (*The effect of T. evansi infestations on plant defenses*), total offspring mortality (unhatched eggs and dead juveniles) was also analyzed and the cbind function was applied. To account for overdispersion, a generalized linear mixed model with a beta-binomial error distribution was used (glmmadmb, glmmADMB package) [57].

To analyze the leaf reflectance factors (*ρ*) on the UV-B spectra (300.4 nm, 303.7 nm, 310.5 nm and 313.9 nm), Box-cox transformations [55] were performed to improve normality when needed (300.4 nm: λ = −3.6; 310.5 nm: λ = −11.5) and linear mixed-effect models (lm, lme4 package) [56] were used.

To analyze the relative content of TIs present in each sample (TIs per total soluble protein content), a Box-cox transformation [55] was performed to improve normality (λ = 0.23) and a linear mixed-effect model (lm, lme4 package) was used [56].

## Supplementary Materials

Supplementary materials can be found at http://www.mdpi.com/1422-0067/19/6/1783/s1.

## Abbreviations

JA	Jasmonic Acid
TI	Trypsin Inhibitors
BApNA	*N*-Benzoyl-d,l-arginin-4 nitroamilide hydrochloride
