# Supplementary material for: Suppression of Plant Defenses by Herbivorous Mites Is Not Associated with Adaptation to Host Plants
Source: Int J Mol Sci. 2018 Jun 15;19(6):1783. doi: 10.3390/ijms19061783 (PMC6032058; doi:10.3390/ijms19061783)
Supplement: Supplementary file 1 [file ijms-19-01783-s001.pdf]

Suplementary Materials

Suppression of Plant Defenses by Herbivorous Mites Is Not Associated with Adaptation to Host Plants

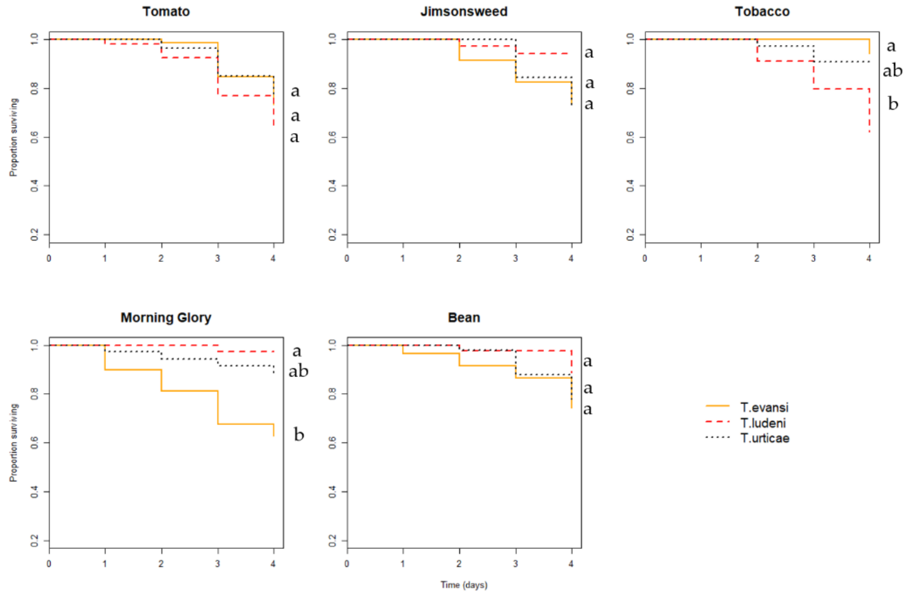

Figure S1. Survival curves of *T. evansi*, *T. ludeni* and *T. urticae* on tomato, jimsonsweed, morning glory or bean leaf discs during 4 days. Different lowercase letters indicate significant differences in survival among mite species within plant species.

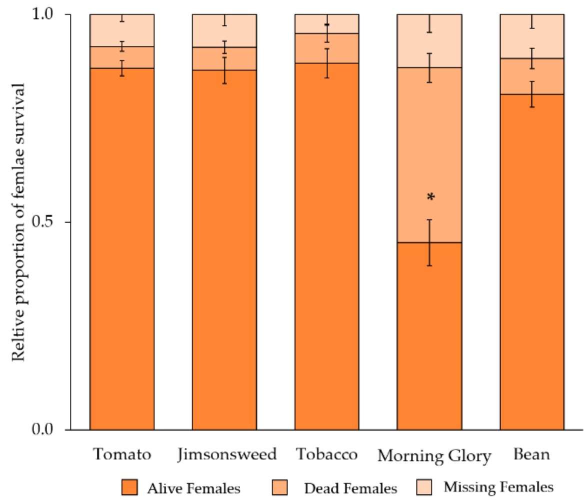

Figure S2. Relative proportion of alive, dead and missing *T. evansi* females after the infestation protocol (bottom to top). The asterisk (*) indicates a significant difference in the proportion of alive females among plant species.

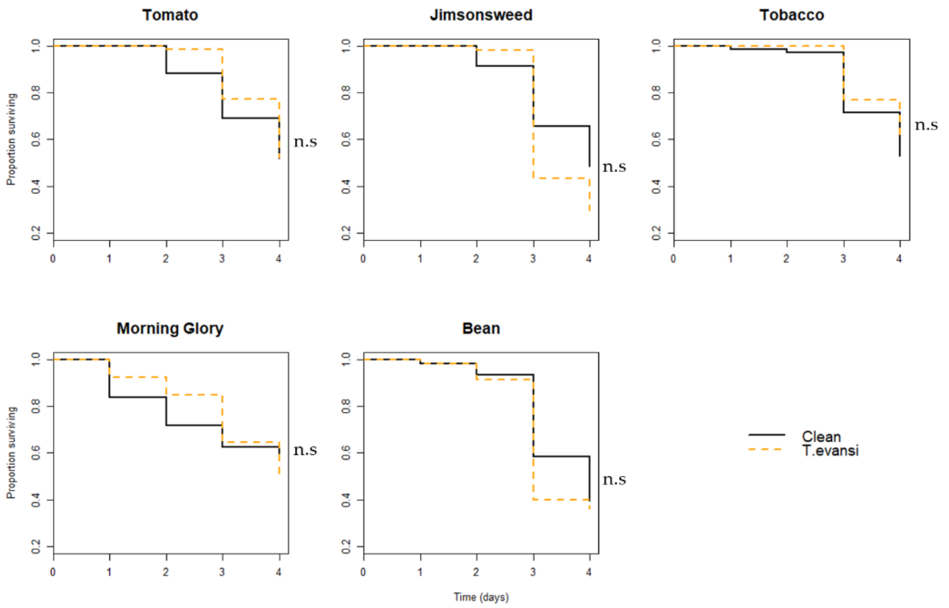

Figure S3. Survival curves of *T. evansi* on tomato, jimsonsweed, morning glory or bean leaf discs, either clean or pre-infested by conspecifics, followed during 4 days. n.s. indicates non-significant differences on daily fecundity among spider mites within plant species.

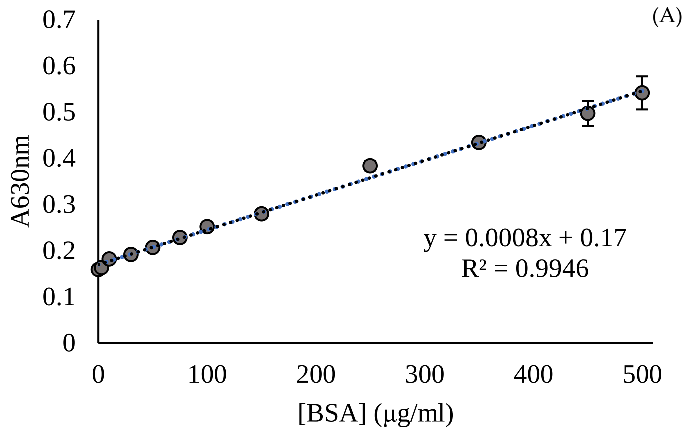

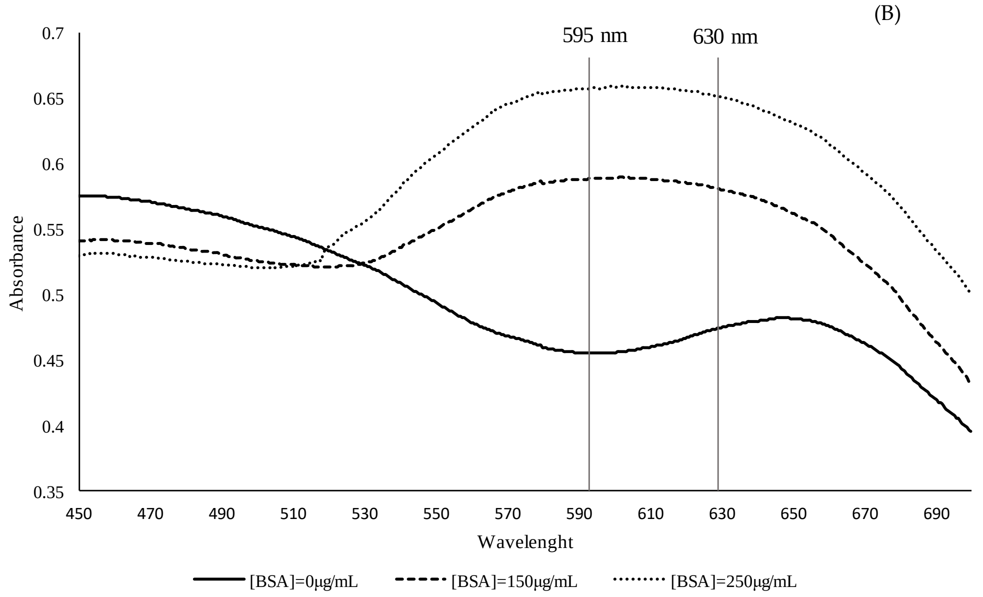

Figure S4. (A) Standard curve for Bradford assay. Markers represent the average (± s.e.) of the three replicated measurements of the known protein content present in the several BSA solutions (μg/mL): 0, 3, 10, 30, 50, 75, 100, 150, 250, 350, 450, 500. (B) Absorbance of three Bradford assays with different concentrations of BSA (0, 150 and 250 μg/mL) in a spectral range of 450 to 700nm. Vertical lines point out the wavelength at which Bradford assay is usually performed (595nm) and the wavelength at which the assay was performed in this project. As represented in figure, the differences in absorbance in the quantification of 150 and 250 μg/mL of protein are minimal.

Table S1. Multiple comparisons using Tukey contrasts (multcomp package)*** $p < 0.001$, * $p < 0.05$.

Variable of Interest	Contrast		Estimate	Std. Error	z value	Pr (>|z|)
**Performance of *Tetranychus* species on several host plants**						
Survival (leaf disc) (day, censor)	Tobacco	*T. ludeni-T. evansi*	2.053	0.778	2.639	0.025*
		T. urticae-T. evansi	0.519	0.913	0.563	1.000
		T. urticae-T. kudeni	-1.534	0.660	-2.322	0.061
	Morning glory	*T. ludeni-T. evansi*	-2.859	1.039	-2.752	0.018*
		T. urticae-T. evansi	-1.249	0.571	-2.189	0.086
		T. urticae-T. kudeni	1.610	1.118	1.440	0.450
Oviposition Rate (fecundity/day)	Tomato	*T. ludeni-T. evansi*	-1.655	0.090	-18.33	<0.001***
		T. urticae-T. evansi	-1.554	0.091	-17.15	<0.001**
		T. urticae-T. kudeni	0.101	0.084	1.209	0.680
	Tobacco	*T. ludeni-T. evansi*	-0.081	0.086	-0.939	1
		T. urticae-T. evansi	-0.605	0.085	-7.127	<0.001***
		T. urticae-T. kudeni	-0.524	0.075	-7.033	<0.001***
	Morning glory	*T. ludeni-T. evansi*	0.992	0.115	8.611	<0.001***
		T. urticae-T. evansi	-0.075	0.116	-0.651	1
		T. urticae-T. kudeni	-1.067	0.111	-9.600	<0.001***
Dead Juveniles (cbind(deadjuv,unhatch+aliveoffspring)	Tomato	*T. ludeni-T. evansi*	3.449	0.446	7.729	<0.001***
		T. urticae-T. evansi	1.281	0.204	6.278	<0.001***
		T. urticae-T. kudeni	-2.168	0.316	-6.856	<0.001***
	Tobacco	*T. ludeni-T. evansi*	-0.668	0.717	-0.931	1.000
		T. urticae-T. evansi	-1.736	0.697	-2.490	0.038*
		T. urticae-T. kudeni	-1.069	0.917	-1.166	0.731
	Morning glory	*T. ludeni-T. evansi*	-2.862	0.439	-6.521	<0.001***
		T. urticae-T. evansi	-2.995	0.466	-6.426	<0.001***
		T. urticae-T. kudeni	-0.133	0.555	-0.239	1.000
**The effect of *T. evansi* infestations on plant defences**						
Survival (infestation) Alive females	Jimsonsweed – Bean		0.695	0.265	2.620	0.088
	Morning glory – Bean		-1.755	0.257	-6.828	<0.001***
	Tobacco – Bean		0.453	0.285	1.588	1.000
	Tomato – Bean		0.389	0.265	1.469	1.000
	Morning glory – Jimsonsweed		-2.450	0.375	-6.525	<0.001***
	Tobacco – Jimsonsweed		-0.241	0.423	-0.571	1.000
	Tomato – Jimsonsweed		-0.306	0.411	-0.744	1.000
	Tobacco - Morning glory		2.208	0.389	5.676	<0.001***
	Tomato – Morning glory		2.144	0.368	5.818	<0.001***
	Tomato - Tobacco		-0.064	0.392	-0.164	1.000

Table S2. Fixed explanatory factors (plant, infestation status and interaction) significance on the several wavelengths used in spectral analysis.

The Effect of *T. Evansi* Infestations on Plant Defences						
**Variable of Interest**		**Fixed explanatory factors**	**Df residuals**	**Df**	**F**	**P**
Reflectance spectroscopy	UV-B (300.4nm)	plant	47.35	4	2.163	0.088
		infestation status	50.10	*1*	*71.65*	<0.001***
		plant*infestation status	42.09	4	1.326	0.276
	UV-B (303.7 nm)	plant	47.11	4	2.344	0.068
		infestation status	50.08	1	50.57	<0.001***
		plant*infestation status	42.07	4	1.415	0.246
	UV-B (307.1 nm)	plant	48.45	4	1.798	0.144
		infestation status	50.28	1	52.43	<0.001***
		plant*infestation status	42.20	4	1.244	0.307
	UV-B (310.5 nm)	plant	47.47	*4*	*3.178*	0.022*
		infestation status	46.09	1	147.1	<0.001***
		plant*infestation status	42.10	4	1.506	0.218
	UV-B (313.9 nm)	plant	47.43	*4*	*2.625*	0.046*
		infestation status	46.10	1	60.08	<0.001***
		plant*infestation status	42.11	4	1.053	0.391

Table S3. Description of the statistical models used in the analysis of the experiments presented in this study.

<table>
<tr><th colspan="2">Variable of Interest</th><th>Response variable</th><th>Data subset</th><th>Sampling size</th><th>Maximal Model</th><th>Minimal Model</th><th>R subroutine</th></tr>
<tr><td colspan="8">Performance of Tetranychus species on several host plants</td></tr>
<tr><td colspan="2" rowspan="3">Survival (leaf disc)</td><td rowspan="3">(day,censor)</td><td>Complete</td><td>695[1]</td><td>plant*spp+(1|block)</td><td>1+(1|block)</td><td rowspan="3">coxme</td></tr>
<tr><td>tomato, jimsonsweed, bean</td><td>181, 103, 155[1]</td><td>spp+(1|block)</td><td>1+(1|block)</td></tr>
<tr><td>tobacco, prurple</td><td>110, 146[1]</td><td>spp+(1|block)</td><td>spp+(1|block)</td></tr>
<tr><td colspan="2" rowspan="3">Oviposition Rate</td><td rowspan="3">fecundity/day</td><td>Complete</td><td>692[1]</td><td>plant*spp+(1|block)</td><td>plant*spp+(1|block)</td><td rowspan="3">lmer[n]
(λ=0.154)</td></tr>
<tr><td>jimsonsweed, bean</td><td>103, 155[1]</td><td>spp+(1|block)</td><td>1+(1|block)</td></tr>
<tr><td>tomato, tobacco, morning glory</td><td>180, 108, 146[1]</td><td>spp+(1|block)</td><td>spp+(1|block)</td></tr>
<tr><td rowspan="4">Relative proportion of offspring</td><td>Embryonic Mortality</td><td>cbind(unhatch,deadjuv+aliveoffspring)</td><td>Complete</td><td>688[1]</td><td>plant*spp+(1|block)</td><td>plant+spp+(1|block)</td><td>glmmadmb[betab]</td></tr>
<tr><td rowspan="3">Dead Juveniles</td><td rowspan="3">cbind(deadjuv,unhatch+aliveoffspring)</td><td>Complete</td><td>688[1]</td><td>plant*spp+(1|block)</td><td>plant*spp+(1|block)</td><td rowspan="3">glmmadmb[betab]+ zeroInflation</td></tr>
<tr><td>jimsonsweed, bean</td><td>103, 154[1]</td><td>spp+(1|block)</td><td>1+(1|block)</td></tr>
<tr><td>tomato, tobacco, morning glory</td><td>178, 108, 145[1]</td><td>spp+(1|block)</td><td>spp+(1|block)</td></tr>
<tr><td colspan="8">The effect of T. evansi infestations on plant defences</td></tr>
<tr><td rowspan="2">Survival (infestation)</td><td>Missing females</td><td>cbind(missing,alive+dead)</td><td>Complete</td><td>36[2]</td><td>plant+(1|block)</td><td>1+(1|block)</td><td>glmmadmb[betab]</td></tr>
<tr><td>Alive females</td><td>cbind(alive,missing+dead)</td><td>Complete</td><td>36[2]</td><td>plant+(1|block)</td><td>plant+(1|block)</td><td>glmmadmb[betab]</td></tr>
<tr><td colspan="2">Survival (leaf disc)</td><td>(day,censor)</td><td>Complete</td><td>663[1]</td><td>plant*infest+(1|block)</td><td>plant+(1|block)</td><td>coxme</td></tr>
<tr><td colspan="2">Oviposition Rate</td><td>fecundity/day</td><td>Complete</td><td>663[1]</td><td>plant*infest+(1|block)</td><td>plant+(1|block)</td><td>lmer[n] (λ=0.265)</td></tr>
<tr><td rowspan="9">Relative proportion of offspring</td><td rowspan="3">Embryonic Mortality</td><td rowspan="3">cbind(unhatch,deadjuv+aliveoffspring)</td><td>Complete</td><td>648[1]</td><td>plant*infest+(1|block)</td><td>plant*infest+(1|block)</td><td rowspan="3">glmmadmb[betab]</td></tr>
<tr><td>jimsonsweed, tobacco, morning glory</td><td>118, 143, 109[1]</td><td>infest+(1|block)</td><td>1+(1|block)</td></tr>
<tr><td>tomato, bean</td><td>157, 121[1]</td><td>infest+(1|block)</td><td>infest+(1|block)</td></tr>
<tr><td rowspan="3">Dead Juveniles</td><td rowspan="3">cbind(deadjuv,unhatch+aliveoffspring)</td><td>Complete</td><td>648[1]</td><td>plant*infest+(1|block)</td><td>plant*infest+(1|block)</td><td rowspan="3">glmmadmb[betab]</td></tr>
<tr><td>jimsonsweed, bean</td><td>118, 121[1]</td><td>infest+(1|block)</td><td>1+(1|block)</td></tr>
<tr><td>tomato, tobacco, morning glory</td><td>157, 143, 109[1]</td><td>infest+(1|block)</td><td>infest+(1|block)</td></tr>
<tr><td rowspan="3">Total Mortality</td><td rowspan="3">cbind(total,aliveoffspring)</td><td>Complete</td><td>648[1]</td><td>plant*infest+(1|block)</td><td>plant*infest+(1|block)</td><td rowspan="3">glmmadmb[betab]</td></tr>
<tr><td>tomato, jimsonsweed</td><td>157, 118[1]</td><td>infest+(1|block)</td><td>1+(1|block)</td></tr>
<tr><td>tobacco, morning glory, bean</td><td>143, 109, 121[1]</td><td>infest+(1|block)</td><td>infest+(1|block)</td></tr>
<tr><td colspan="2" rowspan="2">Reflectance spectroscopy</td><td>ρ303.7, ρ307.1, ρ313.9</td><td>Complete</td><td>57[2]</td><td>plant*infest+(1|block)</td><td>infest+(1|block), infest+(1|block), plant+infest+(1|block)</td><td>lm[n]</td></tr>
<tr><td>ρ300.4, ρ310.5</td><td>Complete</td><td>57[2]</td><td>plant*infest+(1|block)</td><td>infest+(1|block), plant+infest+(1|block)</td><td>lm[n]
(λ= -3.600, λ= -11.50)</td></tr>
<tr><td colspan="2" rowspan="3">Trypsin Inhibitors</td><td rowspan="3">[TIs]</td><td>Complete</td><td>63[2]</td><td>plant*infest+(1|block)</td><td>plant*infest+(1|block)</td><td rowspan="3">lm[n] (λ=0.230)</td></tr>
<tr><td>tomato, jimsonsweed, morning glory, bean</td><td>15, 11, 9, 12[2]</td><td>infest+(1|block)</td><td>1+(1|block)</td></tr>
<tr><td>tobacco</td><td>16[2]</td><td>infest+(1|block)</td><td>infest+(1|block)</td></tr>
</table>

In Cox proportional hazard models (coxme) the response variables include censored individuals (whose death was accidental or by drown). Models with binomial error structure require a concatenated response variable binding together the number of successes and failures for a given outcome. Sampling size gives the number of plants or female mites included in each analysis. "Maximal model" gives the complete set of explanatory variables (and their interactions) included in the model. "Minimal model" gives the model containing only the significant variables and their interactions. Round brackets indicate that the variable was fitted as a random factor. Square brackets indicate the error structure used (n: normal errors, betab: betabinomial errors, coxme does not do predictions); zeroInflation indicates that the model accounts for a high proportion of zeros in the dataset; λ indicates the lambda value used in Box-Cox tranformations. plant: plants species, infest: infestation status (clean vs *T. evansi* pre-infested), spp: spider mite species tested, block: block. Survival (leaf disc)- day: day at which females die; Oviposition Rate - fecundity: number of eggs laid by each females; Relative proportion of offspring - embryonic mortality: number of unhatched eggs (=fecundity-hatch), dead juveniles: number of dead juveniles, total mortality: number of unhatched eggs and dead juveniles (total=unhatch+deadjuv); Trypsin inhibitors: estimate concentration of

TIs given by formula (10); Reflectance spectroscopy: ϱ: Spectral reflectance factors in each wavelength tested. [1]Corresponds to all females used (i.e., the number of leaf discs), [2]Corresponds to all plants used

Table S4. Taxonomic description, age and leaf number of plants used.

Order	Family	Subfamily	Genus	Species	Variety (strain)	Producer	Age (weeks)	Leaf number (from below)
**Solanales**	Solanaceae	Solanoideae	*Solanum*	*lycopersicum*	Moneymaker (*wild-type)*	Johnsons	5	3 or 4
			Datura	*stramonium*		UTAD[1]	5	4
		Nicotianoideae	*Nicotiana*	*tabacum*	Virginia	FCUL[2]	5	6
	Convolvulaceae	Ipomoeeae	*Ipomoea*	*purpurea*	Vigorous	Vilmorin	5	3
**Fabales**	Fabaceae	Faboideae	*Phaseolus*	*vulgaris*	Contender	Germisem	2	1

Seeds kindly provided by: [1]Dr. António Crespi from Botanical Garden of University of Trás-os-Montes e Alto Douro. [2]Dr. Fernando Dias from Faculty of Sciences of University of Lisbon.
